# Quality of life in patients with benign paroxysmal positional vertigo and/or Ménière's disease

**DOI:** 10.1016/S1808-8694(15)31248-9

**Published:** 2015-10-20

**Authors:** Patrícia Rumi Handa, Ana Maria Baccari Kuhn, Fabiana Cunha, Ricardo Schaffleln, Fernando Freitas Ganança

**Affiliations:** Speech Therapist, Master in Sciences, Program of Post-graduation in Human Communication Disorders, Federal University of Sao Paulo - Escola Paulista de Medicina; Psychologist, Psychoanalyst, Full Professor, Head of the Sector of Otoneuropsychology, Department of Otorhinolaryngology and Head and Neck Surgery, Federal University of Sao Paulo - Escola Paulista de Medicina; Speech and Hearing Therapist, Master in Sciences, Program of Post-graduation in Otorhinolaryngology and Head and Neck Surgery, Federal University of Sao Paulo - Escola Paulista de Medicina; Otorhinolaryngologist, Master in Sciences, Program of Otorhinolaryngology and Head and Neck Surgery, Federal University of Sao Paulo - Escola Paulista de Medicina; Otorhinolaryngologist, Ph.D. in Medicine, UNIFESP - EPM, Affiliated Professor, Discipline of Otoneurology, UNIFESP - EPM

**Keywords:** quality of life, vertigo, Ménière's disease, activities of daily living

## Abstract

Patients with benign paroxysmal positional vertigo and/or Ménière's disease relate damages in quality of life.

**Aim:**

To compare the impact of dizziness on quality of life, in patients with benign paroxysmal positional vertigo and/or Ménière's disease, in crisis and out of crisis, and to evaluate the influence of gender, age and impaired semicircular canal.

**Study design:**

clinical with transversal cohort.

**Material and Method:**

The prospective study was realized in 2003/04 at Federal University of São Paulo. The Dizziness Handicap Inventory was applied in seventy patients with positional vertigo, seventy with Ménière's disease and fifteen with both. Two-proportion equality test and the Analysis of variance were employed in this study.

**Results:**

When comparing the groups, Dizziness Handicap Inventory results evidenced higher averages in crisis and out of crisis for Ménière's disease group than for positional vertigo group. The same occurred only during the crisis period in the group when comparing with both disorders (p < 0,05). No significant statistical differences were observed, when comparing the results considering age, gender and, in the group with positional vertigo, affection of posterior semicircular canal as variables.

**Conclusions:**

Ménière's disease patients presented worse quality of life when compared to BPPV patients, in and out of crisis, and during the crisis when regarding the patients with association of both disorders. The damage on quality of life was independent of gender, age and in the BPPV cases it was independent of posterior canal affection.

## INTRODUCTION

Dizziness is one of the most common symptoms in the world, affecting both genders and with higher prevalence in adults, especially the elderly^1^. Vertigo is the most frequent type of dizziness, characterized by the rotation sensation of lack of spatial orientation^2^.

There are many different characteristic otoneurological clinical presentations that may be found in patients with vertigo and other types of dizziness. Ganança et al.^3^ reported that the two most prevalent labyrinthopathies are benign paroxysmal positional vertigo (BPPV) amounting to 19% of the cases, and endolymphatic hydropsis, including Ménière's disease, which affects 17.7% of the patients with dizziness.

BPPV is characterized by onset of rotation dizziness triggered by different positions that the head takes on space, such as neck hyperextension, lateral rotation of head and when the patient lies down or stands up from bed. The dizziness is normally marked and quick, lasts less than one minute and may cause loss of balance and fall^4^.

The recurrent character of clinical manifestations of BPPV frequently causes head movement restrictions to reduce the onset and intensity of vertigo episodes. The perturbation of body balance together with the limitation of movements may impair the performance of daily, school, professional and social activities. Moreover, emotional affections resulting from dizziness may contribute to the deterioration of quality of life of these patients^5^.

Meniere's disease (MD) is characterized by vertigo episodes, which last from 4 to 72 hours on average, hearing loss, tinnitus and ear fullness^6^. The perturbation of spatial disorientation may be significant and normally it is followed by body instability, gait deviation and loss of balance, in addition to neurovegetative symptoms that include general malaise, sudoresis, tachycardia, paleness, spontaneous urination and/or defecation^1^.

The discomfort generated by the symptoms of MD may affect relevantly and permanently the capacity to perform daily activities. The fluctuating character of this labyrinthic disorder, progression of the lesion as a result of clinical progression, unpredictability of future vertigo episodes and emotional affections, determine worsening of quality of life of patients^7^.

Some patients present simultaneous diagnosis of BPPV and MD8., 9., 10.. The association of these labyrinthic disorders in the same subject could cause maximization of the harmful effects of dizziness on quality of life. Some authors^8^ observed that patients with MD associated with BPPV did not respond to therapeutic maneuvers. In such cases, repetitive distensions of the membranous labyrinth caused by endolymphatic hypertension would cause a collapse of the labyrinth or facilitate the adhesion of particles, which would prevent removal of the semicircular canals.

Do patients with BPPV present better or worse quality of life than MD patients? Does association of both labyrinthopathies imply worsened quality of life? Can quality of life impairment be influenced by gender or age range?

The interference of dizziness in quality of life may be assessed by validated questionnaires. Among the existing options, there is the Dizziness Handicap Inventory (DHI)^11^ that assesses the self-perception of the disabling effects imposed by dizziness. In this perspective, we performed the translation, cultural adaptation and assessment of reproducibility of DHI to enable its reliable application to the Brazilian population. The obtained questionnaire was named Brazilian DHI^12^.

We did not find in the scientific literature any studies in which they had compared quality of life and patients with BPPV and MD and the possible influence of gender, age range and in cases of BPPV, semicircular canal affected.

Thus, the present study aimed at comparing the damage caused by dizziness on quality of life of patients with BPPV and/or MD, both during and off crises, the most affected aspects and to assess the influence of gender, age range and affected semicircular canal in BPPV.

## MATERIAL AND METHOD

It was a prospective series study performed under the supervision of the Discipline of Otoneurology, Federal University of Sao Paulo - Escola Paulista de Medicina. The Research Ethics Committee of UNIFESP/Hospital Sao Paulo analyzed and approved the study, whose protocol number is 1491/03.

The study included 155 patients that signed the free informed consent, including 70 patients with BPPV, 70 cases of MD and 15 associations of both.

As to BPPV, it included patients that presented positioning vertigo followed by nystagmus with limited duration, latency and fatigue when repeating the Dix-Hallpike test^13^. Patients with BPPV were classified according to affected semicircular canal, according to the characteristics of the nystagmus^14^.

The exclusion criteria were signs and symptoms of central and auditory impairment (except for cases of presbycusis), affections to vertebral spine or neck, that prevented them from performing the diagnostic or therapeutic maneuver, patients that presented only vertigo when performing the diagnostic maneuver or use of drugs that could interfere in the vestibular study.

The clinical diagnosis of MD was defined in patients that presented spontaneous episodes and recurrent vertigo, sensorineural loss, tinnitus and ear fullness, following the diagnostic criteria set for MD defined by the Hearing and Balance Committee of American Academy of Otorhinolaryngology in 1995^15^.

The patients that presented clinical diagnosis associated with BPPV and MD were included, regardless of the order of installation of the affection, following the same criteria previously mentioned. Patients with other otoneurological disorders did not participate in the study.

We would like to emphasize that these patients that already been treated with anti-vertigo drugs in at least one moment of their clinical progression history.

The patients underwent anamnesis, ENT physical examination and otoneurological assessment that comprised audiological assessment, immittanciometry and vectoelectronystagmography. Electrocochleography was also performed in some patients with diagnostic hypothesis of MD. All patients were characterized according to gender (male and female) and age range (below 41 years, from 41 to 60 years, and above 60).

The instrument that assessed the interference of dizziness on quality of life (QL) was Brazilian DHI applied as an interview to the patients. The questionnaire comprises 25 questions with multiple choice answers yes, no or sometimes. To each affirmative answer, 4 points were scored, negative answers scored zero, and “sometimes” scored 2 points. The maximum total points was 100, and the higher the score, the higher the interference of dizziness in the patients' QL.

The questions are divided into domains that enable different aspects of the life to be assessed. Thus, there are 7 questions that assess physical aspects, 9 emotional aspects and 9 functional aspects.

In cases of BPPV, patients were initially in the crisis, that is, they had positional vertigo and limited duration of nystagmus, latency and fatigue upon repetition of Dix-Hallpike. They all came back to be reassessed one week after the maneuver, up to the moment the patients had no more vertigo or positioning nystagmus. DHI was applied in the crisis and after-crisis period, in which the patients did not present positioning nystagmus any more, after the necessary measures of statoconia repositioning.

Brazilian DHI was applied to patients with MD who, at the time, did not have the crisis, that is, did not necessarily presented all characteristic symptoms, with partial or complete improvement of the disease. As to the period of crisis, we asked them to use the crisis period as a reference to answer the questionnaire.

The patients that presented the association of BPPV and MD answered the questionnaire in the period of BPPV crisis. DHI was also applied after statoconia repositioning maneuvers, with remission of positioning nystagmus characteristic of BPPV.

It was not possible to assess QL in the period of MD crisis, in patients with association of BPPV and MD, because patients referred difficulty to distinguish the aspects that were most affected by the dizziness of BPPV and/or MD.

As to statistical analysis, we used the following techniques: Analysis of Variance (ANOVA), involving quantitative variables, and Two Proportion Similarity Test, for qualitative variables.

In the comparison of physical, emotional and functional aspects in and off-crises, we initially weighted numbers of questions in each aspect: physical – 7 questions, emotional – 9 questions, and functional – 9 questions, and then we applied the ANOVA test.

ANOVA was used in the analysis of Brazilian DHI, checking the possible influence of gender, age range and affected semicircular canal in patients with BPPV.

## RESULTS

In patients with BPPV, MD and association of both, ages ranged respectively from 14 to 82, 22 to 80 and 46 to 80 years. In the classification by age range, 50% of the patients with MD and 60% of those with association of both pathologies were aged from 41 to 60 years, and 54.3% of the patients in the group with BPPV were aged more than 60 years.

In all groups, there were approximately 70% female patients.

As to semicircular canal affection in patients with BPPV, 3 (4.3%) of the patients presented the lateral canal affected, 4 (5.7%) the anterior, 62 (88.6%) the posterior and only 1 (1.4%) patient presented the lateral and posterior canals affected. In cases of associated BPPV and MD, 2 (13.3%) presented the lateral canal affected, 1 (6.7%) the anterior, and 12 (80.0%) the posterior. None of the patients in this group presented more than one affected semicircular canal.

The results obtained with the application of Brazilian DHI were higher in the crises compared to off-crises results in all aspects of the questionnaire, with statistically significant difference (p < 0.05) in the three studied groups.

Upon comparing the QL between the groups with BPPV and MD isolated, we noticed that in all aspects, both in and off-crises, there was statistically significant difference among the groups (Table 1). At both situations, the mean of the MD group was higher than that of the BPPV group.

**Table 1:** Mean, weighted mean, standard deviation and significance of the comparison of physical, emotional, functional and total scores of Brazilian DHI in patients with BPPV and MD, in and off crises.

C	FC Brazilian DHI	BPPV	MD Means	MP	DP	p-value
	Physical	BPPV	17,57	2,51	0,93	0,002*
		MD	20,94	2,99	0,88	
	Emotional	BPPV	15,60	1,73	1,12	< 0,001*
C		MD	22,51	2,50	1,02	
	Functional	BPPV	19,77	2,20	1,09	< 0,001*
		MD	27,57	3,06	0,95	
	Total scores	BPPV	52,89	6,44	2,77	< 0,001*
		MD	71,17	8,56	2,37	
	Physical	BPPV	6,11	0,87	1,02	0,010*
		MD	9,40	1,34	1,09	
	Emotional	BPPV	6,71	0,75	0,96	0,040*
FC		MD	10,03	1,11	1,14	
	Functional	BPPV	7,94	0,88	1,13	0,033*
		MD	11,57	1,29	1,09	
	Total scores	BPPV	20,74	2,50	2,88	0,013*
		MD	31,00	3,74	2,91	

Key: DHI: Dizziness Handicap Inventory, MD: Ménière's disease, BPPV: benign paroxysmal positional vertigo, C: crisis, FC: off-crisis, MD: weighted mean, DP: standard deviation.

However, upon comparing the group with BPPV and the association, we noticed that there was no statistically significant difference between them in physical, emotional, functional and total score values in and off-crises (Table 2).

**Table 2:** Mean, weighted mean, standard deviation and significance of the comparison of physical, emotional, functional and total scores of Brazilian DHI in patients with BPPV and association of BPPV and MD, in and off crises.

C	FC Brazilian DHI	BPPV	MD Means	MP	DP	p-value
	Physical	BPPV	17,57	2,51	0,93	0,446
		BPPV and MD	18,93	2,70	0,66	
	Emotional	BPPV	15,60	1,73	1,12	0,784
C		BPPV and MD	14,80	1,64	1,20	
	Functional	BPPV	19,77	2,20	1,09	0,699
		BPPV and MD	18,67	2,07	1,22	
	Total scores	BPPV	52,89	6,44	2,77	0,878
		BPPV and MD	51,87	6,42	2,66	
	Physical	BPPV	6,11	0,87	1,02	0,889
		BPPV and MD	6,40	0,91	1,04	
	Emotional	BPPV	6,71	0,75	0,96	0,857
FC		BPPV and MD	6,27	0,70	1,02	
	Functional	BPPV	7,94	0,88	1,13	0,722
		BPPV and MD	6,93	0,77	0,99	
	Total scores	BPPV	20,74	2,50	2,88	0,868
		BPPV and MD	19,60	2,38	2,90	

**Key**: DHI: Dizziness Handicap Inventory, MD: Ménière's disease, BPPV: benign paroxysmal positional vertigo, C: crisis, FC: off-crisis, MD: weighted mean, DP: standard deviation.

In the comparison of patients with MD and association of BPPV and MD, we detected that only in the crises situations there was statistically significant difference, in emotional, functional and total scores, indicating higher scores for the group with MD only (Table 3).

**Table 3:** Mean, weighted mean, standard deviation and significance of the comparison of physical, emotional, functional and total scores of Brazilian DHI in patients with MD and association BPPV and MD in and off crises.

C	FC Brazilian DHI	BPPV	MD Means	MP	DP	p-valor
	Physical	DM	20,94	2,99	0,88	0,236
		BPPV and MD	18,93	2,70	0,66	
	Emotional	DM	22,51	2,50	1,02	0,005*
C		BPPV and MD	14,80	1,64	1,20	
	Functional	DM	27,57	3,06	0,95	0,001*
		BPPV and MD	18,67	2,07	1,22	
	Total scores	DM	71,17	8,56	2,37	0,001*
		BPPV and MD	51,87	6,42	2,66	
	Physical	DM	9,40	1,34	1,09	0,169
		BPPV and MD	6,40	0,91	1,04	
	Emotional	DM	10,03	1,11	1,14	0,193
FC		BPPV and MD	6,27	0,70	1,02	
	Functional	DM	11,57	1,29	1,09	0,094
		BPPV and MD	6,93	0,77	0,99	
	Total scores	DM	31,00	3,74	2,91	0,104
		BPPV and MD	19,60	2,38	2,90	

**Key:** DHI: Dizziness Handicap Inventory, MD: Ménière's disease, BPPV: benign paroxysmal positional vertigo, C: crisis, FC: off-crisis, MD: weighted mean, DP: standard deviation.

As to the aspect assessed by Brazilian DHI in the group with BPPV, the statistically significant difference (p < 0.05) was also present between them in the periods of crises, and the most affected one was the physical aspect, followed by the functional one, and finally, the emotional one. Off-crises, there was no statistically significant difference between these three aspects (p = 0.685).

In the group with MD, the physical and functional aspects presented higher scores in crises and there was no statistically significant difference between both (p = 0.643). In this group, there was no statistically significant difference between the emotional and functional aspects (p = 0.341). Off-crises, in the group with MD, there was no statistically significant difference between the aspects (p = 0.447). The same was observed in the group with BPPV and MD off-crises (p = 0.837).

Upon correlating the scores of physical, emotional, functional and total scores of Brazilian DHI by gender and age range of patients we did not observe mean statistically significant difference in any of the studied groups in and off-crises periods.

In the comparison of results of Brazilian DHI in groups with BPPV and association of BPPV and MD, both with posterior semicircular canal affection, we detected no statistically significant difference between the groups, both in crises and off them. The same analysis was not performed with the other semicircular canals (superior and lateral), because the sample was not enough and it was not possible to compare the damage of dizziness in QL between the different affected canals.

## DISCUSSION

Dizziness is one of the most important symptoms with negative influence in the wellbeing of subjects of both genders and different age ranges. It is present in different vestibular system affections, among which we can include BPPV and MD, considered as the most prevalent in many different studies9., 10.^,^^16^, which originated the present investigation.

Brazilian DHI was the instrument chosen to determine the harmful effects caused by dizziness in the studied population because it is a specific and unique questionnaire, translated and adapted to the Brazilian population, easy to apply and to understand.

The findings of Brazilian DHI in the three groups were greater in the period of crises, because it is at this moment that there is exacerbation of dizziness. QL damage was also seen off-crisis in most studied patients, which shows the disabling character of BPPV, MD and their association.

In patients with isolated BPPV, we detected improvement of QL after statoconia repositioning maneuver showing the effectiveness of this therapeutic option. Similar results were also found in other research studies^5^^,^17., 18. that used DHI-S and Brazilian DHI as assessment instrument.

Upon comparing the scores of the questionnaire during the isolated BPPV crises, physical aspects were the most affected, followed in decreasing order by functional and emotional aspects. These findings are partially in accordance with other study with BPPV^19^, which observed more marked damage in physical and functional aspects. The treatment of choice did not produce statistically significant difference between the analyzed off-crises aspects, showing improvement in QL obtained in all aspects studied by DHI.

The temporary character of dizziness in BPPV and the situations in which this symptom can occur could justify the greater impairment of physical aspects seen in the DHI. After the crisis, which last some seconds, patients reported they could perform their daily living activities. This fact could explain why those physical aspects were more affected than functional ones.

The emotional aspect was less affected because the abnormalities to this element are normally resultant from physical limitations and long lasting functional disabilities caused by dizziness that affect professional, social and family life.

In isolated MD, Brazilian DHI was applied in the period off-crisis, requiring patients to report initially to this moment and later to the crisis, because not all patients with MD presented crisis while the study was being performed.

We also noticed greater damage in physical and functional aspects, which is in accordance with other study with MD^20^. The damage resulting from dizziness in MD, especially in these two aspects, may be explained by the chronic character of the disease, with fluctuating, recurrent and long lasting clinical manifestations that may impair not only the physical capabilities, but also the activities of the subjects.

In the association of BPPV and MD, the most affected aspect in the situation of crises was the physical aspect. This result was already expected given that in this group, the patients were assessed in the period of crises with BPPV.

The application of Brazilian DHI in the studied population showed that dizziness has harmful influence on QL of patients in all dimensions of the daily life.

The assessment of physical aspects enabled the verification between eye movement, head and body and onset or worsening of dizziness.

The emotional aspect allowed the assessment of the presence of frustration, fear of going out alone or staying home alone, embarrassment of the clinical manifestations of the disease, concern about self-image, difficulty to concentrate, sensation of incapacity, depression and problems related with family and social issues and dizziness.

In the study by Grimby, Rosenhall^21^, subjects with dizziness reported more frequent problems of memory, anxiety, panic disorder and agoraphobia, than subjects without dizziness. Other studies reported the strong correlation between dizziness and emotional affections. Many different authors^22^^,^^23^ emphasized the fear of new vertigo attacks, increase in distress and phobias as a result of the labyrinthopaties.

The functional aspect enabled the confirmation of damage to performing professional, home, social, leisure activities in addition to assessing the dependence to perform some specific tasks, such as walking with help and difficulty to walk at home in the dark.

In correlating data of Brazilian DHI with different clinical pictures in this study, the results showed that patients with isolated MD, in situations of crises and off-crises, presented statistical means higher than patients with BPPV, indicating worse QL owing to dizziness.

In cases of BPPV, significant improvement and reduction of disability caused by it in these patients should be due to the benign character of positional vertigo, application of appropriate treatment, in addition to possible spontaneous remission^4^.

MD, in turn, has higher variability of triggering and aggravating agents and clinical manifestations that may be persistent and fluctuating, requiring more prolonged treatment and sometimes the association of many different therapeutic options - drugs, food reeducation, vestibular rehabilitation, psychotherapy and surgical procedures^6^.

The association of BPPV and MD may influence the results of treatment with BPPV, worsening the therapeutic responses to statoconia repositioning maneuvers^8^.

Despite this, results show that BPPV associated with MD did not influence QL when compared to isolated BPPV in and off-crises. This finding showed that symptomatology of MD off-crises was no sufficiently intense to worsen the damage to QL in patients with both labyrinthopathies.

In the same study, upon comparing the period of crises in patients with MD, and the period of crises with BPPV in patients with both vestibular dysfunctions, we noticed that QL was the most affected one in the group with isolated MD. Such finding may be justified by the fact that the patients with both labyrinthopathies were assessed in the period of crises of BPPV, but in the intercritical phase of MD.

We did not find in the literature any result of DHI applied in periods in and off-crises in patients with associated BPPV and MD, preventing the comparison of data.

Upon correlating the results of Brazilian DHI and gender, there was no influence of this variable in the three groups, indicating that gender does not have a relevant impact on QL. The same finding was also noticed in other studies about MD^20^^,^^24^ and BPPV^25^. However, upon correlating the data of DHI and results of computed dynamic posturography in patients with vestibular pathologies in general, women had higher score in all subscales of DHI^26^.

Upon correlating the results of Brazilian DHI and age, we detected that there was no influence of age range in the damage caused by dizziness on QL in and off-crisis.

Age has not influenced the results in the application of this questionnaire in the study of QL in MD^20^ and BPPV^25^. Despite the aging of body balance mechanisms, the higher likelihood of chronic-degenerative disease and the use of multiple drugs, among other factors, which may favor the onset of dizziness or aggravate its intensity^12^, the impact of dizziness on QL occurred in all studied age ranges, without significant difference.

As to affection of posterior semicircular canal, in the patients with BPPV and the association of both vestibular dysfunctions, there was no difference in affection to QL between the two groups, indicating that clinical manifestations in the intercritical period of MD were not marked enough to aggravate the harm to QL of patients with the association.

We did not find scientific evidence referring to semicircular canals of patients with BPPV associated with MD relating them to the results obtained in the application of Brazilian DHI.

Studies with Brazilian DHI or other questionnaires, specific or nonspecific, should be developed to expand the knowledge about self-perception of damage caused by dizziness in patients with BPPV and/or MD, both in the period in and off-crisis, checking the clinical progression of patients submitted to different available therapeutic options.

## CONCLUSIONS

1. Patients with BPPV, MD and their association presented affected QL owing to dizziness in relation to physical, functional and emotional aspects assessed by the Brazilian DHI in the periods in and off-crises and the QL affection was higher in the crisis period.
2. All patients with MD presented worsened QL owing to dizziness when compared to BPPV in all aspects and in relation to the association only of functional and emotional aspects in the crises of BPPV.
3. Physical aspects were more affected in the group with associated BPPV and MD, whereas physical and functional aspects were more affected in the MD group.
4. The QL affection was independent of gender, age range and in cases of BPPV and the association, there was no influence of posterior semicircular canal.
